# 

**Published:** 1892-01

**Authors:** 


### CONTRIBUTORS TO VOL. XXII.

T. Hilliard Wood, M. D., Nashville, Tennessee.
C. D. Roy, A. B., M. D., New York.
Andrew Boyd, M. D., Scottsboro, Ala.
E. Van Goidtsnoven, M. D., Atlanta, Ga.
Llewellen P. Barber, M. D., Tracy City, Tenn.
Sidney B. Wright, Chattanooga, Tenn.
C. H. Holland, M. D., Chattanooga, Tennessee.
P. O'Keef, M. D., Oconto, Wis.
C. A. Gill, M. D., Madison, Wis.
A. H. Currier, M. D, New York.
T. L. Lallerstadt, M. D., Panola, Ga.
R. O. Cotter, M. D., Macon, Ga.
A. B. Patterson, M. D., Atlanta, Ga.
E. H. M. Parham, M. D., Fordyce, Ark.
R. J. Mitchell, M. D., Girard, Ill.
J. McFadden Gaston, M. D., Atlanta, Ga.
E. H. Root, M. D., Chicago, Ill.
W. A. Crow, M. D., Atlanta, Ga.
Thomas D. Coleman, A. M., M. D, Augusta, Ga.
E. H. Richardson, M. D., Atlanta, Ga.
A. S. Johnson, M. D., Bowman, Ga.
W. H. Doughty, M. D., Augusta, Ga.
T. M. Holmes, M. D., Rome, Ga.
Jno. H. McIntyre, A M., M. D., St. Louis, Mo.
J. W. Duncan, M. D., Atlanta, Ga.
L. B. Anderson, M. D., Norfolk, Va.
Emory Lamphear, M. A., M. D., Kansas City, Mo.
A. J. Swaney, M. D., Gellatin, Tenn.
Karl Von Ruck, B. S., M. D., Asheville, N. C.
H. McHatton, M. D., Macon, Ga.
E. S. McKee, M. D., Cincinnati, O.
L. G. Hardman, M. D., Harmony Grove, Ga.
A. C. Davidson, M. D., Sharon, Ga.
W. B. Cox, M. D., Landsford, S. C.
Hugh Hagan, M. D., Atlanta, Ga.
W. S. Gottheil, M. D., New York.
R. R. Kime, M. D., Atlanta, Ga.
S. C. Carson, M. D., Bessemer, Ala.
P. McCahey, M. D., Philadelphia.
G. Wiley Broome, M. D., St. Louis, Mo.
E. H. Camp, M. D., Gadsden, Ala.
Geo. R. West, M. D., Chattanooga, Tenn.
J. A. Childs, M. D., Atlanta, Ga.
Wm. Perrin Nicolson, M. D, Atlanta, Ga.
N. O. Harris, M. D., Atlanta, Ga.
L. H. Jones, M. D., Atlanta, Ga.
L. B. Grandy, M. D., Atlanta, Ga.
J. C. Johnson, M. D., Atlanta, Ga.
F. O. Stockton, M. D., Atlanta, Ga.
W. C. Jarnagin, M. D., Atlanta, Ga.
C. G. Giddings, M. D., Atlanta, Ga.
N. B. Warren, M. D, Hazel Dell, Miss.
Bransford Lewis, M. D., Vienna.
T. M. McIntosh, M. D., Paris.
P. J. Rosenheim, M. D., New York.

